# Supplementary material for: Satellite forecasting of crop harvest can trigger a cross-hemispheric production response and improve global food security
Source: Commun Earth Environ. 2023 Sep 21;4(1):334. doi: 10.1038/s43247-023-00992-2 (PMC11041643; doi:10.1038/s43247-023-00992-2)
Supplement: Supplementary file 2 — Supplementary Information [file 43247_2023_992_MOESM2_ESM.pdf]

Supplementary Information for

Remote-sensing forecasting of harvest can trigger a cross-hemispheric supply responses and improve global food security

Tetsuji Tanaka,[1,2] Laixiang Sun,[2,3,*] Inbal Becker-Reshef,[2] Xiao-Peng Song,[2] Estefania Puricelli,[2]

[1] Department of Economics, Meiiji Gakuin University, 1-2-37 Shirokanedai Minato-ku Tokyo, 108-8636, Japan

[2] Department of Geographical Sciences, University of Maryland, College Park, MD 20471, USA.

[3] School of Finance & Management, SOAS University of London, Russell Square, London WC1H 0XG, UK.

[*] Corresponding author: Laixiang Sun.

E-mail: LSun123@umd.edu, Tel. +1 301 4058131.

Supplementary Note 1. Supplementary Tables to the Main Results

Supplementary Note 2. Elasticities Employed and Sensitivity Analysis

Supplementary Note 3. Supplementary Tables to the Method Section

Supplementary Note 4. Creating the soybean sector in the SAM

Supplementary Note 5. World trade Computable General Equilibrium (CGE) model

Supplementary Note 1. Supplementary Tables to the Main Results

Supplementary Table 1. Forecasting of Wheat Production (million tons) in Russia & Ukraine in 2008 and 2012: A comparison

	2008			2012		
	Real prod.	Our RS forecasting	WASDE forecasting	Real prod	Our RS forecasting	WASDE forecasting
	89.7			53.5		
May		80.1	72.0		66.2	76.0
June		79.5	75.0		62.6	74.0

Note: The changes were relative to the production level in the previous year.

Source: Historical records of wheat production are from FAO (2021). The forecasting data of the USDA-WASDE are from USDA (2023).

Supplementary Table 2. Farmers' responses in Northern Hemisphere under the S-April12 scenario

	Change in production [%]			Change in production [ton]
	Soybean	Wheat	Other Grains	Soybean
United States	4.84	-0.17	-0.03	4,080,901
Canada	7.40	-0.14	-0.02	330,614
Total				4,411,514.6

Supplementary Table 3. Changes in CIF prices of soybean by major importers under the S-Real12 and S-April12 scenario

	S-Real12	S-April12
China	15.4	2.5
Taiwan (customs territory)	15.3	1.7
Germany	14.6	2.1
Indonesia	14.2	-3.8
Japan	14.9	-4.5
Mexico	15.9	-6.4
Netherland	15.0	2.8
Spain	15.5	8.3
Thailand	15.3	7.0
Africa	13.6	-1.0
Asia	14.0	1.3

Supplementary Note 2. Elasticities Employed and Sensitivity Analysis

Supplementary Table 4 The elasticities assumed in the main scenarios

	Sector	Armington composite	Substitution of production factors		Substitution of food products
			2007 model	2011 model	
1	Paddy Rice	2.53	0.24	0.25	*
2	Wheat	2.23	0.24	0.25	*
3	Other Grains	0.65	0.24	0.25	*
4	Vegetable and Fruit	0.93	0.24	0.25	*
5	Oilseeds	1.23	0.24	0.25	*
6	Soybean**	1.23	0.24	0.25	*
7	Sugar Cane and Beet	1.35	0.24	0.25	*
8	Plant Fiber	2.50	0.24	0.25	
9	Other Crops	1.63	0.24	0.25	*
10	Meat and Livestock	1.58	0.51	0.50	*
11	Processed Food	1.06	1.12	1.12	*
12	Transport	1.90	1.68	1.68	
13	Service	1.95	1.36	1.36	
14	Others	3.61	1.03	1.00	

Notes: the asterisks indicate the elasticity of substitution of household between food-related goods, which is set as 0.2 following Seale et al. (2003). The double asterisk means that the sector "Soybean" is embedded only with the CGE model for the soybean analysis. Following Bajzik et al. (2020), the Armington elasticities in the GTAP-v10 are halved for food-related sectors (i.e., sectors 1, 2, 3, 4, 5, 6, 7, 9, 10, and 11) because our analysis focuses on short-term impacts of farmers' response to remote sensing forecasting information. The substitution elasticities of production factors are taken from the GTAP-v10 database. We run an additional set of sensitivity tests by halving these substitution elasticities of production factors for food-related sectors as we do for the Armington elasticities, and the results are presented in Tables S7, S8, S15, S16, S21, and S22.

Supplementary Table 5. Sensitivity analysis for Armington elasticity ±30%: Changes in real local wheat prices in the 2008 and 2012 scenarios (%)

	Change in real wheat price [%]					
	Armington +30%			Armington –30%		
	W-Real08	W-May08	W-June08	W-Real08	W-May08	W-June08
Bangladesh	-36.3	-32.7	-32.8	-33.6	-30.4	-30.6
China	-30.5	-22.1	-22.5	-22.6	-16.0	-16.3
Egypt	-36.3	-34.0	-34.1	-33.4	-31.7	-31.8
India	-35.4	-33.2	-33.3	-31.7	-29.9	-30.0
Japan	-30.7	-22.4	-22.8	-25.0	-18.0	-18.3
Korea	-30.0	-20.3	-20.8	-24.0	-15.7	-16.1
Nigeria	-29.9	-23.3	-23.6	-24.0	-18.5	-18.8
Turkey	-32.3	-30.8	-30.9	-27.0	-26.1	-26.2
Middle East	-33.6	-30.2	-30.4	-29.1	-26.4	-26.6
Africa	-30.0	-25.6	-25.8	-24.3	-20.8	-21.0
	Armington +30%			Armington –30%		
	W-Real12	W-May12	W-June12	W-Real12	W-May12	W-June12
Bangladesh	25.2	15.7	12.8	20.2	13.2	11.0
China	22.1	13.1	10.4	15.4	9.4	7.5
Egypt	32.3	24.9	22.6	28.9	24.1	22.6
India	14.7	9.1	7.5	8.1	5.3	4.5
Japan	26.4	16.5	13.6	20.9	13.7	11.4
Korea	26.4	15.6	12.5	20.9	12.8	10.3
Nigeria	25.0	16.6	14.1	19.2	13.6	11.8
Turkey	32.5	26.4	24.5	28.8	25.3	24.1
Middle East	26.7	18.5	16.0	21.5	16.1	14.3
Africa	25.6	17.9	15.6	20.2	15.4	13.8

Supplementary Table 6. Sensitivity analysis for Armington elasticity ±30%: Changes in real local soybean prices in the 2012 scenarios (%)

	Change in real soybean price [%]			
	Armington +30%		Armington −30%	
	S-Real12	S-April12	S-Real12	S-April12
China	15.4	2.4	15.4	2.4
Taiwan (customs territory)	15.3	1.7	15.3	1.7
Germany	14.6	2.1	14.6	2.1
Indonesia	13.8	-3.7	13.8	-3.7
Japan	14.9	-4.5	14.9	-4.5
Mexico	15.9	-6.4	15.9	-6.4
Netherland	15.0	2.8	15.0	2.8
Spain	15.5	8.3	15.5	8.3
Thailand	15.2	7.0	15.2	7.0
Africa	12.2	-0.4	12.2	-0.4
Asia	12.8	1.0	12.8	1.0

Supplementary Table 7. Sensitivity analysis for the elasticity of substitution between the factors of production with the variations by ±30% and that for food-related sectors with the variation by –50%: Changes in real local wheat prices in the 2008 and 2012 scenarios (%)

	Change in real wheat price [%]								
	Value added +30%			Value added –30%			Value added (food) –50%		
	W-Real08	W-May08	W-June08	W-Real08	W-May08	W-June08	W-Real08	W-May08	W-June08
Bangladesh	-34.9	-31.5	-31.6	-35.6	-32.2	-32.3	-36.0	-32.5	-32.7
China	-27.1	-19.3	-19.7	-27.7	-19.9	-20.3	-28.0	-20.2	-20.6
Egypt	-34.9	-32.8	-32.9	-35.6	-33.5	-33.6	-36.0	-33.9	-34.0
India	-33.7	-31.7	-31.8	-34.4	-32.3	-32.4	-34.7	-32.7	-32.8
Japan	-28.2	-20.3	-20.7	-28.8	-20.9	-21.3	-29.1	-21.3	-21.7
Korea	-27.4	-18.1	-18.6	-28.0	-18.7	-19.2	-28.3	-19.0	-19.5
Nigeria	-27.3	-21.0	-21.4	-27.9	-21.7	-22.0	-28.2	-22.0	-22.3
Turkey	-29.9	-28.8	-28.9	-30.6	-29.4	-29.4	-30.9	-29.7	-29.7
Middle East	-31.6	-28.5	-28.6	-32.2	-29.1	-29.3	-32.6	-29.5	-29.6
Africa	-27.5	-23.4	-23.6	-28.1	-24.0	-24.2	-28.4	-24.4	-24.6
	Value added +30%			Value added –30%			Value added (food) –50%		
	W-Real12	W-May12	W-June12	W-Real12	W-May12	W-June12	W-Real12	W-May12	W-June12
Bangladesh	22.5	14.1	11.5	24.0	15.5	12.9	24.8	16.4	13.8
China	18.7	11.1	8.7	19.9	12.2	9.9	20.6	13.0	10.7
Egypt	30.1	23.9	22.0	32.1	25.7	23.7	33.3	26.8	24.8
India	11.3	7.1	5.8	12.0	7.8	6.5	12.5	8.3	7.0
Japan	23.5	14.7	12.1	25.0	16.2	13.5	25.9	17.2	14.5
Korea	23.4	13.8	10.9	25.0	15.3	12.4	25.9	16.3	13.4
Nigeria	22.0	14.8	12.6	23.4	16.2	13.9	24.3	17.0	14.8
Turkey	30.1	25.3	23.8	32.2	27.1	25.5	33.4	28.2	26.6
Middle East	23.9	16.9	14.8	25.5	18.4	16.2	26.4	19.3	17.1
Africa	22.7	16.4	14.4	24.2	17.7	15.7	25.1	18.5	16.5

Supplementary Table 8. Sensitivity analysis for the elasticity of substitution between the factors of production with the variations by ±30% and that for food-related sectors with the variation by –50%: Changes in real local soybean prices in the 2012 scenarios (%)

	Change in real soybean price [%]					
	Value added +30%		Value added –30%		Value added (food) –50%	
	S-Real12	S-April12	S-Real12	S-April12	S-Real12	S-April12
China	15.4	2.4	15.4	2.4	16.8	2.7
Taiwan (customs territory)	15.3	1.7	15.3	1.7	16.6	2.0
Germany	14.6	2.1	14.7	2.1	15.9	2.3
Indonesia	13.8	-3.7	13.8	-3.7	15.0	-3.5
Japan	14.9	-4.5	14.9	-4.5	16.2	-4.5
Mexico	15.9	-6.4	15.9	-6.4	17.3	-6.4
Netherland	15.0	2.8	15.0	2.8	16.3	3.0
Spain	15.5	8.3	15.5	8.3	16.9	8.7
Thailand	15.2	7.0	15.2	7.0	16.5	7.3
Africa	12.2	-0.4	12.2	-0.4	13.3	-0.2
Asia	12.8	1.0	12.8	1.0	13.9	1.1

Supplementary Table 9. Sensitivity analysis for the elasticity of substitution between food commodities for household ±30%: Changes in real local wheat prices in the 2008 and 2012 scenarios (%)

	Change in real wheat price [%]					
	Food +30%			Food –30%		
	W-Real08	W-May08	W-June08	W-Real08	W-May08	W-June08
Bangladesh	-34.0	-31.0	-31.2	-36.8	-32.7	-32.9
China	-23.8	-17.2	-17.6	-31.9	-22.4	-22.9
Egypt	-33.8	-32.1	-32.2	-36.9	-34.4	-34.5
India	-32.2	-30.5	-30.6	-36.1	-33.6	-33.8
Japan	-25.9	-19.0	-19.4	-31.6	-22.5	-23.0
Korea	-25.0	-16.8	-17.2	-31.0	-20.3	-20.8
Nigeria	-24.9	-19.5	-19.8	-30.9	-23.5	-23.9
Turkey	-27.9	-27.0	-27.0	-33.1	-31.5	-31.6
Middle East	-29.9	-27.3	-27.4	-34.4	-30.5	-30.7
Africa	-25.4	-22.0	-22.1	-30.8	-25.8	-26.1
	Food +30%			Food –30%		
	W-Real12	W-May12	W-June12	W-Real12	W-May12	W-June12
Bangladesh	19.3	12.6	10.5	28.9	17.7	14.3
China	15.2	9.3	7.5	25.6	14.9	11.7
Egypt	27.2	22.3	20.8	36.5	28.1	25.4
India	8.3	5.5	4.6	17.3	10.7	8.7
Japan	20.1	13.1	11.0	30.2	18.6	15.1
Korea	20.1	12.3	10.0	30.2	17.6	13.8
Nigeria	18.6	13.0	11.3	28.7	18.9	15.9
Turkey	27.2	23.4	22.2	36.7	29.8	27.6
Middle East	20.6	15.2	13.5	30.6	21.0	18.0
Africa	19.4	14.5	13.0	29.3	20.5	17.7

Supplementary Table 10. Sensitivity analysis for the elasticity of substitution between food commodities for household ±30%: Changes in real local soybean prices in the 2012 scenarios (%)

	Change in real soybean price [%]			
	Food +30%		Food –30%	
	S-Real12	S-April12	S-Real12	S-April12
China	15.2	2.5	15.2	2.4
Taiwan (customs territory)	15.1	1.8	15.1	1.7
Germany	14.3	2.1	14.3	2.0
Indonesia	13.3	-3.6	13.3	-3.8
Japan	14.6	-4.4	14.6	-4.6
Mexico	15.6	-6.3	15.6	-6.6
Netherland	14.7	2.8	14.7	2.7
Spain	15.4	8.3	15.4	8.2
Thailand	14.9	6.9	14.9	7.0
Africa	11.3	-0.3	11.3	-0.5
Asia	12.1	1.0	12.1	0.9

Supplementary Table 11. Sensitivity analysis for the elasticity of land transformation between crops: Changes in real local wheat prices in the 2008 and 2012 scenarios (%)

	Change in real wheat price in 2008 and 2012 [%]					
	Land +30%			Land –30%		
	W-Real08	W-May08	W-June08	W-Real12	W-May12	W-June12
Bangladesh	-34.8	-31.3	-31.5	-35.8	-32.4	-32.5
China	-27.0	-19.2	-19.6	-27.9	-20.0	-20.4
Egypt	-34.8	-32.7	-32.8	-35.8	-33.7	-33.8
India	-33.5	-31.6	-31.7	-34.6	-32.5	-32.6
Japan	-28.1	-20.2	-20.6	-29.0	-21.1	-21.5
Korea	-27.3	-18.0	-18.5	-28.1	-18.8	-19.3
Nigeria	-27.2	-21.0	-21.3	-28.1	-21.8	-22.1
Turkey	-29.8	-28.7	-28.7	-30.8	-29.6	-29.6
Middle East	-31.5	-28.4	-28.5	-32.5	-29.3	-29.5
Africa	-27.4	-23.3	-23.5	-28.3	-24.2	-24.4

	Land +30%			Land –30%		
	W-Real12	W-May12	W-June12	W-Real12	W-May12	W-June12
Bangladesh	22.5	14.1	11.6	24.1	15.4	12.8
China	18.7	11.1	8.8	20.0	12.2	9.8
Egypt	30.1	24.0	22.0	32.2	25.7	23.7
India	11.3	7.1	5.9	12.1	7.8	6.5
Japan	23.5	14.8	12.2	25.1	16.2	13.4
Korea	23.4	13.9	11.0	25.1	15.3	12.3
Nigeria	22.0	14.9	12.7	23.5	16.1	13.9
Turkey	30.1	25.4	23.8	32.3	27.1	25.5
Middle East	23.9	17.0	14.8	25.5	18.3	16.1
Africa	22.7	16.4	14.5	24.3	17.7	15.7

Supplementary Table 12. Sensitivity analysis for the elasticity of land transformation between crops: Changes in real local soybean prices in the 2012 scenarios (%)

	Change in real soybean price [%]			
	Land +30%		Land –30%	
	S-Real12	S-April12	S-Real12	S-April12
China	15.0	2.4	16.0	2.6
Taiwan (customs territory)	14.9	1.6	15.9	1.8
Germany	14.3	2.0	15.2	2.2
Indonesia	13.5	-3.7	14.3	-3.6
Japan	14.5	-4.5	15.4	-4.5
Mexico	15.5	-6.4	16.5	-6.4
Netherland	14.6	2.7	15.6	2.9
Spain	15.1	8.1	16.1	8.5
Thailand	14.8	6.8	15.7	7.1
Africa	11.9	-0.5	12.7	-0.3
Asia	12.5	0.9	13.3	1.0

Supplementary Table 13. Sensitivity analysis for the Armington elasticity ±30%: Changes in household consumption in the 2008 and 2012 scenarios (%)

	Change in household wheat consumption [%]					
	Armington +30%			Armington –30%		
	W-Real08	W-May08	W-June08	W-Real08	W-May08	W-June08
Bangladesh	9.5	8.3	8.3	8.6	7.6	7.6
China	7.6	5.1	5.2	5.3	3.5	3.6
Egypt	10.2	9.4	9.4	9.2	8.6	8.6
India	9.2	8.5	8.5	8.0	7.5	7.5
Japan	7.6	5.2	5.3	5.9	4.0	4.1
Korea	7.4	4.6	4.7	5.6	3.5	3.6
Nigeria	7.5	5.5	5.6	5.7	4.2	4.3
Turkey	8.3	7.9	7.9	6.7	6.4	6.4
Middle East	8.7	7.6	7.7	7.3	6.5	6.5
Africa	7.7	6.3	6.4	5.9	5.0	5.0
	Armington +30%			Armington –30%		
	W-Real12	W-May12	W-June12	W-Real12	W-May12	W-June12
Bangladesh	-4.4	-2.9	-2.4	-3.6	-2.5	-2.1
China	-3.9	-2.4	-2.0	-2.8	-1.8	-1.4
Egypt	-5.9	-4.7	-4.4	-5.4	-4.6	-4.4
India	-2.7	-1.7	-1.4	-1.5	-1.0	-0.9
Japan	-4.6	-3.0	-2.5	-3.7	-2.5	-2.1
Korea	-4.5	-2.8	-2.3	-3.7	-2.4	-1.9
Nigeria	-4.4	-3.1	-2.6	-3.5	-2.5	-2.2
Turkey	-5.7	-4.8	-4.5	-5.2	-4.6	-4.4
Middle East	-4.8	-3.5	-3.0	-4.0	-3.1	-2.8
Africa	-4.6	-3.4	-3.0	-3.8	-2.9	-2.7

Supplementary Table 14. Sensitivity analysis for the Armington elasticity ±30%: Changes in household soybean consumption in 2012 scenarios (%)

	Change in household soybean consumption [%]			
	Armington +30%		Armington −30%	
	S-Real12	S-April12	S-Real12	S-April12
China	-2.8	-0.5	-2.8	-0.5
Taiwan (customs territory)	-2.8	-0.4	-2.8	-0.4
Germany	-2.7	-0.4	-2.7	-0.4
Indonesia	-2.6	0.7	-2.6	0.7
Japan	-2.7	0.9	-2.7	0.9
Mexico	-2.9	1.3	-2.9	1.3
Netherland	-2.8	-0.6	-2.8	-0.6
Spain	-2.9	-1.6	-2.9	-1.6
Thailand	-2.8	-1.4	-2.8	-1.4
Africa	-2.3	0.1	-2.3	0.1
Asia	-2.4	-0.2	-2.4	-0.2

Supplementary Table 15. Sensitivity analysis for the elasticity of substitution between the factors of production with the variations by ±30% and that for food-related sectors with the variation by –50%: Changes in household consumption in the 2008 and 2012 scenarios (%)

	Change in household wheat consumption [%]								
	Value added +30%			Value added –30%			Value added (food) –50%		
	W-Real08	W-May08	W-June08	W-Real08	W-May08	W-June08	W-Real08	W-May08	W-June08
Bangladesh	9.0	7.9	7.9	9.3	8.1	8.2	9.4	8.2	8.3
China	6.5	4.4	4.5	6.7	4.5	4.6	6.8	4.6	4.7
Egypt	9.7	9.0	9.0	9.9	9.2	9.3	10.1	9.3	9.4
India	8.7	8.0	8.0	8.9	8.2	8.3	9.0	8.3	8.4
Japan	6.8	4.6	4.7	7.0	4.8	4.9	7.1	4.9	5.0
Korea	6.6	4.0	4.2	6.7	4.2	4.3	6.8	4.3	4.4
Nigeria	6.7	4.9	5.0	6.8	5.1	5.2	6.9	5.2	5.2
Turkey	7.6	7.2	7.2	7.8	7.4	7.4	7.9	7.5	7.5
Middle East	8.1	7.1	7.1	8.3	7.3	7.3	8.4	7.4	7.5
Africa	6.9	5.7	5.8	7.1	5.9	5.9	7.2	6.0	6.0
	Value added +30%			Value added –30%			Value added (food) –50%		
	W-Real12	W-May12	W-June12	W-Real12	W-May12	W-June12	W-Real12	W-May12	W-June12
Bangladesh	-4.0	-2.6	-2.2	-4.2	-2.8	-2.4	-4.4	-3.0	-2.6
China	-3.4	-2.1	-1.7	-3.6	-2.3	-1.9	-3.7	-2.4	-2.0
Egypt	-5.6	-4.6	-4.3	-5.9	-4.9	-4.6	-6.1	-5.1	-4.7
India	-2.1	-1.4	-1.1	-2.3	-1.5	-1.3	-2.3	-1.6	-1.4
Japan	-4.1	-2.7	-2.3	-4.4	-3.0	-2.5	-4.5	-3.1	-2.7
Korea	-4.1	-2.5	-2.0	-4.3	-2.8	-2.3	-4.5	-3.0	-2.5
Nigeria	-3.9	-2.8	-2.4	-4.2	-3.0	-2.6	-4.3	-3.1	-2.8
Turkey	-5.4	-4.6	-4.4	-5.7	-4.9	-4.6	-5.9	-5.1	-4.8
Middle East	-4.4	-3.2	-2.8	-4.6	-3.5	-3.1	-4.8	-3.6	-3.2
Africa	-4.2	-3.1	-2.8	-4.4	-3.3	-3.0	-4.6	-3.5	-3.1

Supplementary Table 16. Sensitivity analysis for the elasticity of substitution between the factors of production with the variations by ±30% and that for food-related sectors with the variation by –50%: Changes in household soybean consumption in the 2012 scenarios (%)

	Change in household soybean consumption [%]					
	Value added +30%		Value added –30%		Value added (food) –50%	
	S-Real12	S-April12	S-Real12	S-April12	S-Real12	S-April12
China	-2.8	-0.5	-2.8	-0.5	-3.1	-0.5
Taiwan (customs territory)	-2.8	-0.4	-2.8	-0.4	-3.1	-0.4
Germany	-2.7	-0.4	-2.7	-0.4	-2.9	-0.5
Indonesia	-2.6	0.7	-2.6	0.7	-2.8	0.7
Japan	-2.7	0.9	-2.7	0.9	-3.0	0.9
Mexico	-2.9	1.3	-2.9	1.3	-3.1	1.3
Netherland	-2.8	-0.6	-2.8	-0.6	-3.0	-0.6
Spain	-2.9	-1.6	-2.9	-1.6	-3.1	-1.7
Thailand	-2.8	-1.4	-2.8	-1.4	-3.1	-1.5
Africa	-2.3	0.1	-2.3	0.1	-2.5	0.0
Asia	-2.4	-0.2	-2.4	-0.2	-2.6	-0.3

Supplementary Table 17. Sensitivity analysis for the elasticity of substitution between food commodities for household ±30%: Change in household consumption in the 2008 and 2012 scenarios (%)

	Change in household wheat consumption [%]					
	Food +30%			Food −30%		
	W-Real08	W-May08	W-June08	W-Real08	W-May08	W-June08
Bangladesh	11.5	10.2	10.3	6.7	5.7	5.8
China	7.3	5.0	5.2	5.5	3.6	3.7
Egypt	12.1	11.4	11.4	7.3	6.7	6.7
India	10.7	10.0	10.1	6.5	6.0	6.0
Japan	8.1	5.6	5.8	5.5	3.6	3.7
Korea	7.7	4.9	5.0	5.3	3.2	3.3
Nigeria	7.8	5.9	6.0	5.4	3.9	3.9
Turkey	9.1	8.7	8.7	6.0	5.6	5.6
Middle East	9.9	8.8	8.9	6.2	5.4	5.4
Africa	8.2	6.9	7.0	5.5	4.5	4.5
	Food +30%			Food −30%		
	W-Real12	W-May12	W-June12	W-Real12	W-May12	W-June12
Bangladesh	-4.5	-3.1	-2.6	-3.5	-2.3	-1.9
China	-3.6	-2.3	-1.9	-3.1	-1.9	-1.5
Egypt	-6.5	-5.5	-5.1	-4.8	-3.8	-3.5
India	-2.1	-1.4	-1.2	-2.2	-1.4	-1.2
Japan	-4.7	-3.2	-2.7	-3.6	-2.4	-1.9
Korea	-4.6	-3.0	-2.4	-3.6	-2.2	-1.8
Nigeria	-4.4	-3.2	-2.8	-3.5	-2.4	-2.1
Turkey	-6.3	-5.5	-5.3	-4.5	-3.8	-3.5
Middle East	-4.9	-3.7	-3.3	-3.9	-2.8	-2.4
Africa	-4.7	-3.6	-3.2	-3.7	-2.7	-2.4

Supplementary Table 18. Sensitivity analysis for the elasticity of substitution between food commodities for household ±30%: Change in household soybean consumption in 2012 scenarios (%)

	Change in household soybean consumption [%]			
	Food +30%		Food –30%	
	S-Real12	S-April12	S-Real12	S-April12
China	-3.6	-0.7	-2.0	-0.3
Taiwan (customs territory)	-3.6	-0.5	-2.0	-0.2
Germany	-3.4	-0.6	-2.0	-0.3
Indonesia	-3.2	0.9	-1.9	0.5
Japan	-3.5	1.2	-2.0	0.7
Mexico	-3.7	1.7	-2.1	0.9
Netherland	-3.5	-0.8	-2.0	-0.4
Spain	-3.7	-2.1	-2.0	-1.1
Thailand	-3.6	-1.8	-2.1	-1.0
Africa	-2.8	0.1	-1.7	0.1
Asia	-2.9	-0.3	-1.8	-0.1

Supplementary Table 19. Sensitivity analysis for the elasticity of land allocation ±30%: Change in household wheat consumption in 2008 and 2012 scenarios (%)

	Change in household wheat consumption [%]					
	Land +30%			Land –30%		
	W-Real08	W-May08	W-June08	W-Real08	W-May08	W-June08
Bangladesh	9.0	7.9	7.9	9.3	8.2	8.2
China	6.5	4.4	4.5	6.8	4.6	4.7
Egypt	9.6	8.9	9.0	10.0	9.3	9.3
India	8.6	8.0	8.0	9.0	8.3	8.3
Japan	6.8	4.6	4.7	7.1	4.8	5.0
Korea	6.5	4.0	4.2	6.8	4.2	4.4
Nigeria	6.6	4.9	5.0	6.9	5.1	5.2
Turkey	7.5	7.2	7.2	7.8	7.5	7.5
Middle East	8.0	7.1	7.1	8.3	7.4	7.4
Africa	6.9	5.7	5.7	7.1	5.9	6.0
	Land +30%			Land –30%		
	W-Real12	W-May12	W-June12	W-Real12	W-May12	W-June12
Bangladesh	-4.0	-2.6	-2.2	-4.2	-2.8	-2.4
China	-3.4	-2.1	-1.7	-3.6	-2.3	-1.9
Egypt	-5.6	-4.6	-4.3	-5.9	-4.9	-4.5
India	-2.1	-1.4	-1.1	-2.3	-1.5	-1.3
Japan	-4.1	-2.7	-2.3	-4.4	-2.9	-2.5
Korea	-4.1	-2.6	-2.1	-4.3	-2.8	-2.3
Nigeria	-3.9	-2.8	-2.4	-4.2	-3.0	-2.6
Turkey	-5.4	-4.6	-4.4	-5.7	-4.9	-4.6
Middle East	-4.4	-3.2	-2.8	-4.6	-3.4	-3.1
Africa	-4.2	-3.1	-2.8	-4.4	-3.3	-3.0

Supplementary Table 20. Sensitivity analysis for the elasticity of land allocation ±30%: Change in household soybean consumption in 2012 scenarios (%)

	Change in household soybean consumption [%]			
	Land +30%		Land –30%	
	S-Real12	S-April12	S-Real12	S-April12
China	-2.8	-0.5	-2.9	-0.5
Taiwan (customs territory)	-2.8	-0.3	-2.9	-0.4
Germany	-2.7	-0.4	-2.8	-0.5
Indonesia	-2.5	0.8	-2.7	0.7
Japan	-2.7	0.9	-2.8	0.9
Mexico	-2.8	1.3	-3.0	1.3
Netherland	-2.7	-0.6	-2.9	-0.6
Spain	-2.8	-1.6	-3.0	-1.6
Thailand	-2.8	-1.4	-2.9	-1.4
Africa	-2.3	0.1	-2.4	0.1
Asia	-2.4	-0.2	-2.5	-0.2

Supplementary Table 21. Sensitivity analysis for the elasticities of the Armington, value-added and household food consumption: total wheat production in the Southern Hemisphere (Argentina, Australia, Paraguay, South Africa and Uruguay) (tons)

	Aggregated responsive wheat production in the SH [tons]			
	2008 May	2008 June	2012 May	2012 June
Armington+30%	-3,664,504	-3,500,949	3,220,154	4,258,251
Armington−30%	-1,918,804	-1,833,021	1,881,246	2,529,397
Value added+30%	-2,844,860	-2,718,091	2,638,716	3,514,945
Value added−30%	-2,804,306	-2,678,383	2,558,484	3,407,460
Value added (food)−50%	-2,781,253	-2,655,825	2,488,523	3,312,033
Food+30%	-2,624,600	-2,504,920	2,387,368	3,172,760
Food−30%	-3,073,924	-2,939,940	2,877,948	3,845,180
Land+30%	-2,827,662	-2,701,415	2,615,776	3,483,670
Land−30%	-2,831,492	-2,704,809	2,592,711	3,454,749

Supplementary Table 22. Sensitivity analysis for the elasticities of the Armington, value-added and household food consumption: total soybean production in the Northern Hemisphere (the United States and Canada) (tons)

	Changes in soybean production in NH [tons]
	2012 April
Armington+30%	4,411,664
Armington-30%	4,411,322
Value added+30%	4,413,577
Value added-30%	4,408,697
Value added (food) -50%	4,453,299
Food+30%	4,388,615
Food-30%	4,436,979
Land+30%	4,386,481
Land-30%	4,449,717

Supplementary Note 3. Supplementary Figures and Tables to the Method Section

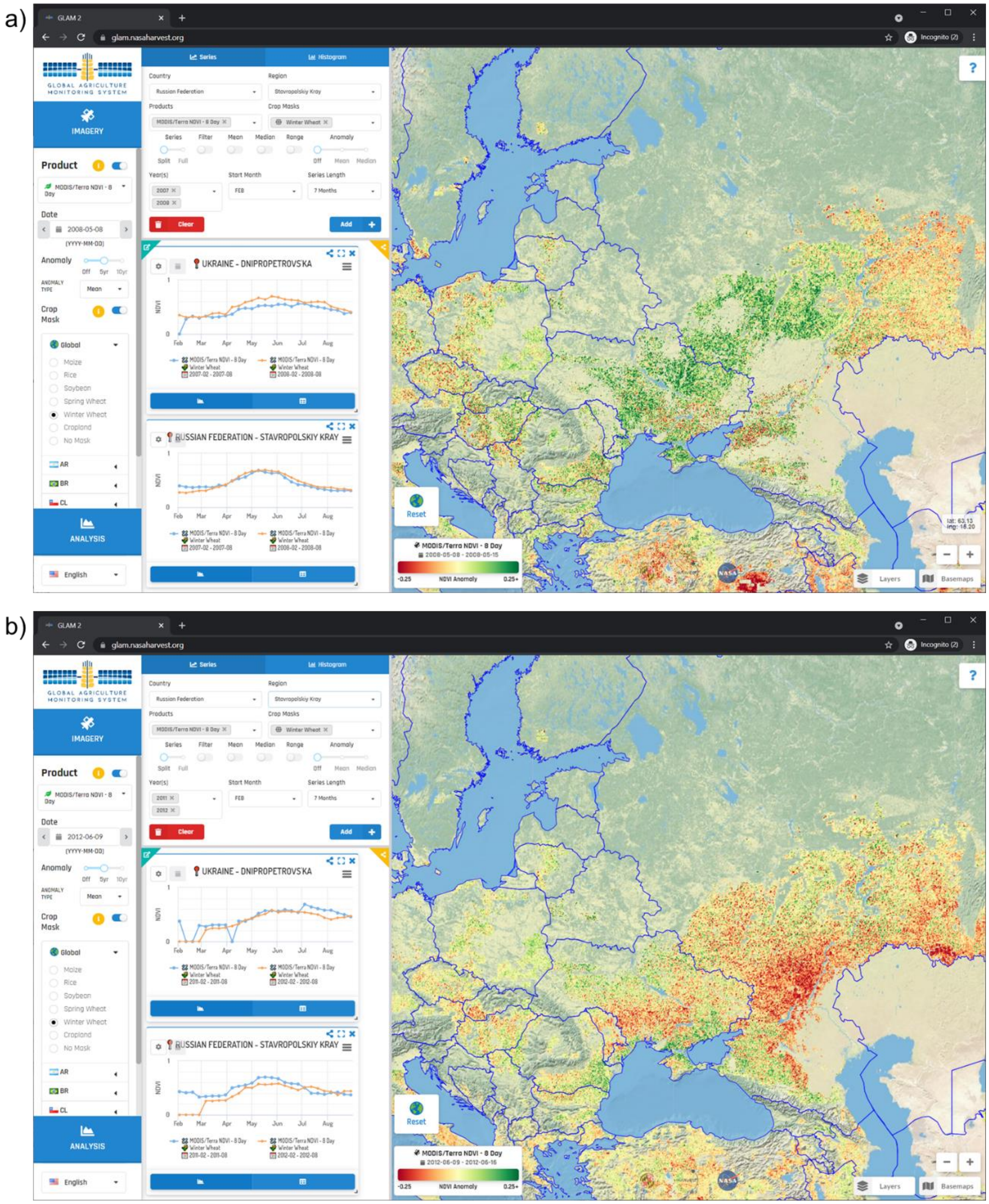

Supplementary Figure 1: Normalized difference vegetation index (NDVI) anomaly during critical winter growing season in wheat producing regions of Ukraine and Russia. a) Positive NDVI anomaly in 2008; b) Negative NDVI anomaly in 2012. In both panels, the line charts on the

left show 8-day NDVI curves of the current year (i.e. 2008 and 2012, respectively) as compared to the previous year, and the maps on the right show NDVI anomalies of the current year as compared to mean NDVI of the previous 5 years. Screenshots were taken from the Global Agriculture Monitoring System (GLAM) at https://glam.nasaharvest.org/.

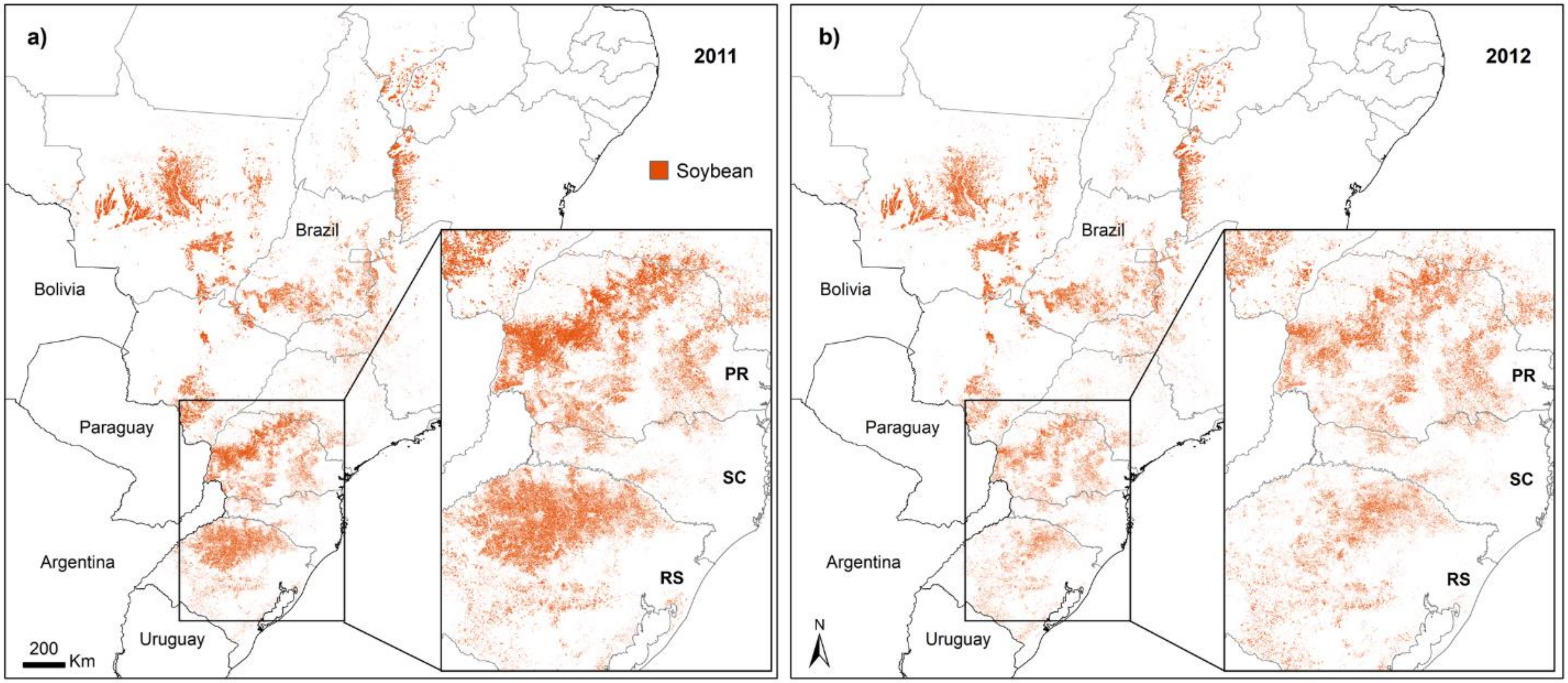

Supplementary Figure 2: Drought-induced soybean production anomaly in southern Brazil in 2012 derived from satellite remote sensing data. a) soybean in 2011; b) soybean in 2012. PR: Parana, SC: Santa Catarina, RS: Rio Grande do Sul.

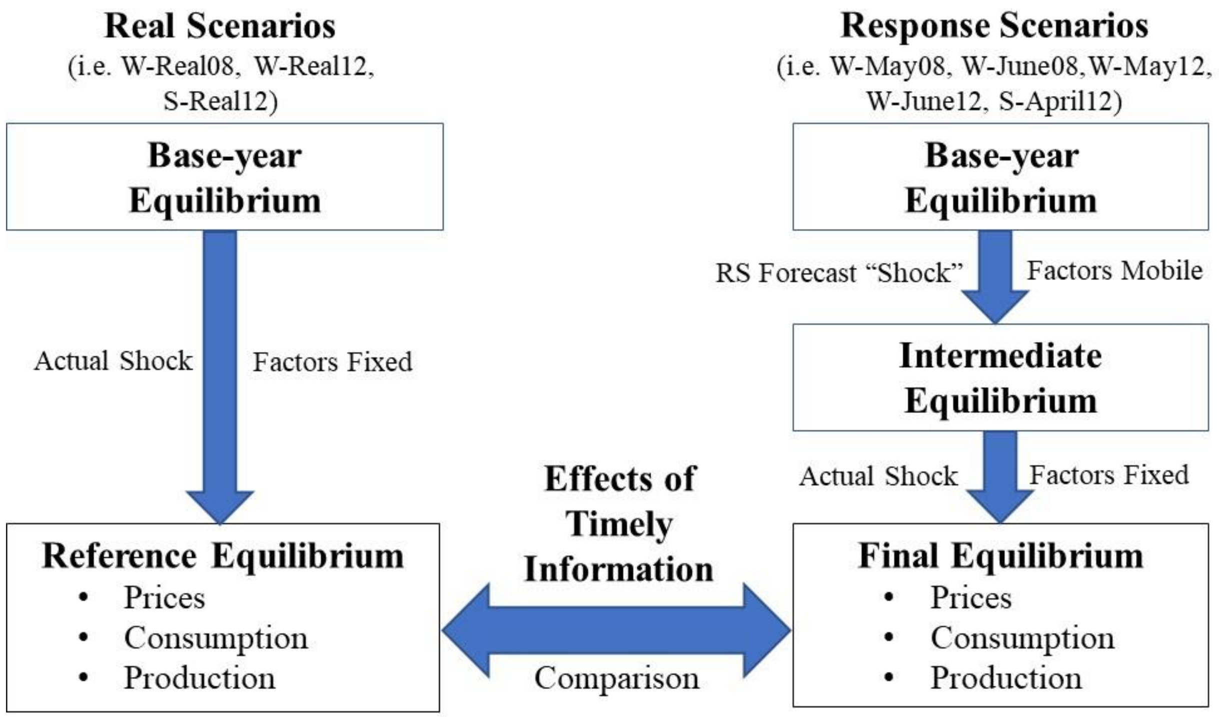

Supplementary Figure 3: The implementation procedure of the hemisphere-wise response estimation

Supplementary Table 23. Shocks, settings and scenarios

		Intermediate Equilibrium				Final Equilibrium		
		Remote sensing forecast				Actual yield shock	Actual yield shock	Experimental
	Crop	April	May	June	Factor mobility	in Russia and Ukraine	in Brazil	Year
W-Base07	Wheat							2007
W-Real08	Wheat					Yes		2008
W-May08	Wheat		Yes		Yes	Yes		2008
W-June08	Wheat			Yes	Yes	Yes		2008
W-Base11	Wheat							2011
W-Real12	Wheat					Yes		2012
W-May12	Wheat		Yes		Yes	Yes		2012
W-June12	Wheat			Yes	Yes	Yes		2012
S-Base11	Soybean							2011
S-Real12	Soybean						Yes	2012
S-April12	Soybean	Yes			Yes		Yes	2012

Supplementary Table 24. Regional and sectoral aggregations of the model for wheat market analysis in 2008 and 2012

Region	Sector
Argentina	Paddy Rice
Australia	Wheat
Bangladesh	Other Grain
Brazil	Vegetable and Fruit
China	Oilseeds
Egypt	Sugar Cane and Beet
Indonesia	Plant Fiber
India	Other Crops
Japan	Meat and Livestock
South Korea	Processed Food
Nigeria	Transport
Philippines	Service
Paraguay	Others
Russia+Ukraine	
Turkey	
Uruguay	
United States of America	
South Africa	
Latin America	
Former Soviet Union	
Europe	
Asia	
Middle East	
Africa	
Rest of the World	

Supplementary Table 25. Regional and sectoral aggregations of the model for soybean market analysis in 2012

Region	Sector
Argentina	Paddy Rice
Brazil	Wheat
Canada	Other Grain
China	Vegetable and Fruit
Taiwan (customs territory)	Soybean
Germany	Other Oil Crops
Indonesia	Sugar Cane and Beet
Japan	Plant Fiber
Mexico	Other Crops
Netherland	Meat and Livestock
Spain	Processed Food
Thailand	Transport
Ukraine	Service
United States	Others
Africa	
Europe	
Asia	
Latin America	
Rest of the World	

Supplementary Note 4. Creating soybean sector in the SAM

The GTAP database version 10 does not explicitly have the soybean sector but contains it in the oilseeds sector (i.e. the sector of the "osd"). We used the Splitcom to extract soybean sector from the original oilseeds sector based on numerical data from the FAOSTAT. The detailed description of the Splitcom package is provided on the website: https://www.gtap.agecon.purdue.edu/resources/splitcom.asp. For splitting the "osd" sector between soybean and other oilseeds sectors, we estimated the production, consumption, import and export ratios of soybean to the other oilseeds crops for the 19 regions in the model (Supplementary Table 21).

Supplementary Note 5. World trade Computable General Equilibrium (CGE) model

Supplementary Figures 4 and 5 presents the major structure of our CGE models while Supplementary Figure 6 reports the land supply structure and the elasticity values in the associated constant-elasticity-of-transformation (CET) functions. In our CGE models, we assume a representative producer in a given sector of a given region maximizes her/his profit under the Leontief technology for producing gross output by using intermediate inputs and a value-added composite, the latter of which is a constant-elasticity-of-substitution (CES) aggregation of production factors with the elasticity of substitution quoted from the GTAP database. Produced goods are allocated between aggregated exports and domestic goods with a CET function. Domestic goods are combined with aggregated imports to generate composite goods under the CES form, as assumed by Armington (1969). Composite imports consist of imports from individual foreign regions, and similarly, composite exports are distributed to individual recipient regions (Supplementary Figures 4-5).

The Armington elasticities are parameters to represent the resemblance of goods or services between regions. Our model does not explicitly consider different types of wheat, but the share parameters in the CES/CET functions for international trade that are calibrated based on historical trade flow, approximately describing the preference of each region. Exchange rates are assumed to be exogenous, while foreign saving is assumed to be endogenous that equates the balance of payments. The saving-driven investment is adopted as a model closure.

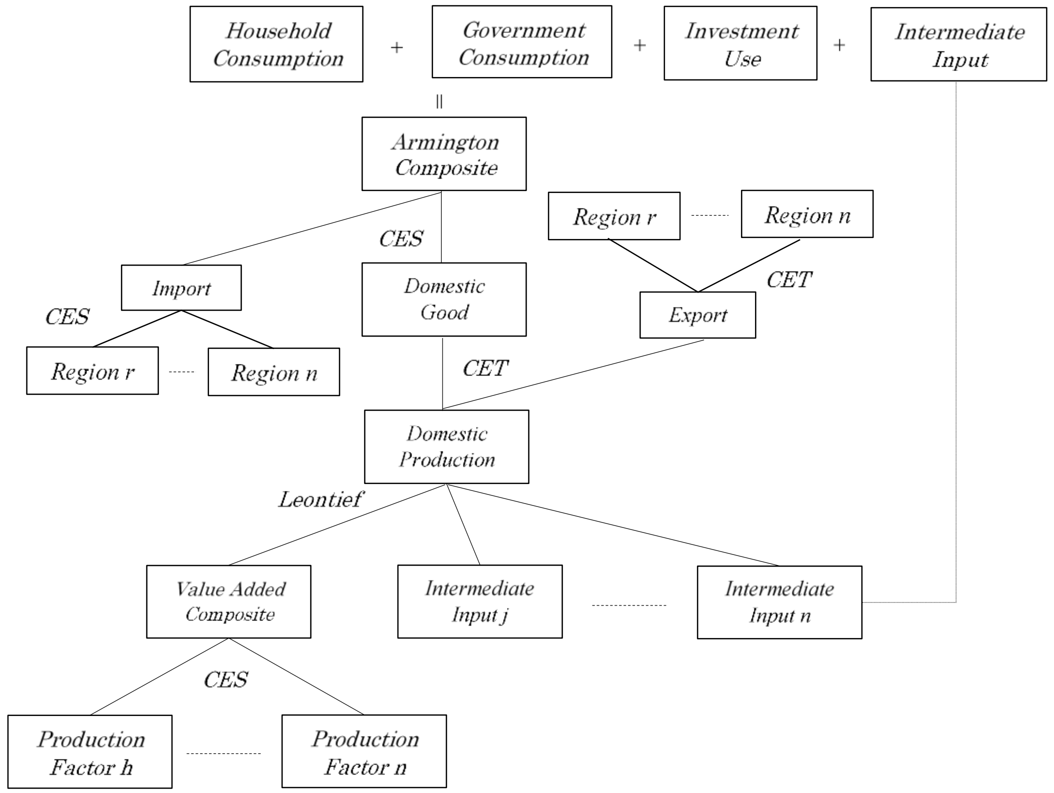

Supplementary Figure 4. Overview of the model structure

Composite goods are consumed by a representative household, government, investment agent, and other sectors (intermediate inputs) in a given region. In the structure of household consumption, food-related commodities are aggregated to generate food composite goods using a CES function, which directly contributes to utility level together with non-food items. The elasticity for food

composite follows the estimates of Seale et al. (2003) (Supplementary Figure 5). The specification for land allocation and the associated elasticity values following Haile et al. (2016) and Timilsina et al. (2012) ((Supplementary Figure 6).

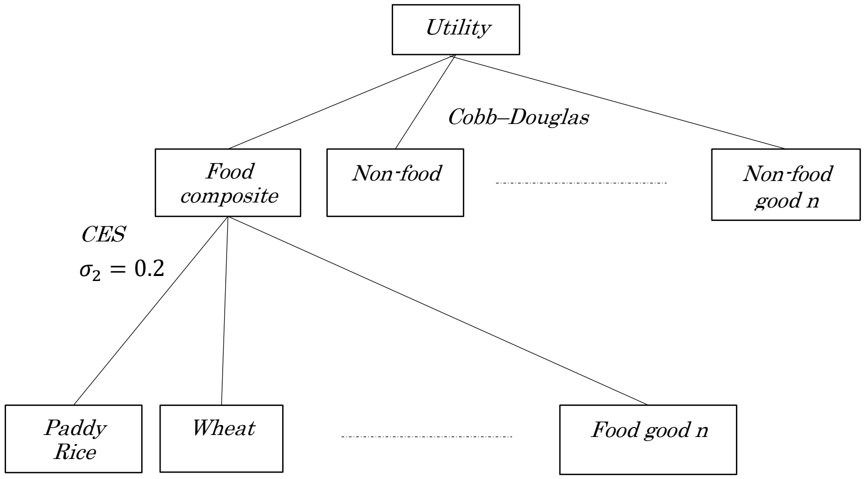

Supplementary Figure 5. Household consumption structure.

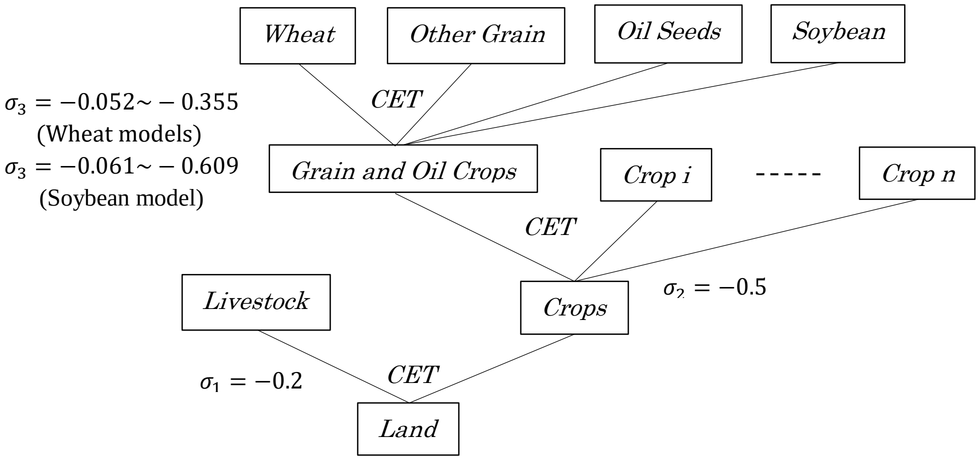

Supplementary Figure 6. Land supply structure and elasticity values in the CET functions

A full specification of our world trade CGE is as follows.

- Symbol

Sets

i, j: commodities/sectors (other than the food composite)

fd: food commodities/sectors

nfd: non-food commodities/sectors

ifd: non-food commodities plus the food composite

jog: oil crops and grain

r, s, r': regions

r_sh: responding regions

h: factors (capital, skilled labor, unskilled labor, farmland, natural resources)

Endogenous variables

$X^p_{i,r}$: household consumption

XFD_r: food composite

$X^g_{i,r}$: government consumption

$X^v_{i,r}$: investment uses

$X_{i,j,r}$: intermediate uses of the *i*-th good by the *j*-th sector

$F_{h,j,r}$: factor uses

$Y_{j,r}$: value added

$Z_{j,r}$: gross output

$Q_{i,r}$: Armington composite good

$M_{i,r}$: composite imports

$D_{i,r}$: domestic goods

$E_{i,r}$: composite exports

$T_{i,r,s}$: inter-regional transportation from the *r*-th region to the *s*-th region

TT_r: exports of inter-regional shipping service by the *r*-th region

Q^s: composite inter-regional shipping service

S_r^p: household savings

S_r^g: government savings

T_r^d: direct taxes

$T_{j,r}^z$: production taxes

$T_{j,s,r}^m$: import tariffs

$T_{j,r,s}^e$: export taxes

$T_{h,j,r}^f$: factor input taxes

$LAND_{r\_sh}$: composite farm land

LSK_{r_sh}: livestock

CRP_{r_sh}: crops

OGC_{r_sh}: oil crop and grain composite

$OCRP_{j\_oc,r\_sh}$: non-oil crop and grain crops

$p_{r\_sh}^{lsk}$: price of livestock

$p_{r_{sh}}^{ld}$: price of farm land

$p_{r\_sh}^{crp}$: price of crops

$p_{r_{sh}}^{ogc}$: price of non-oil and grain crop composite

p_{j_oc,r_sh}^{ocrp}: price of oil crops and grain

p_r^{XFD}: price of food composite

$p_{i,r}^q$: price of Armington composite goods

$p_{h,j,r}^f$: price of factors

$p_{j,r}^y$: price of value added

$p_{i,r}^z$: price of gross output

$p_{i,r}^m$: price of composite imports

$p_{i,r}^d$: price of domestic goods

$p_{i,r}^e$: price of composite exports

$p_{i,r,s}^t$: price of goods shipped from the *r*-th region to the *s*-th region

p^s: inter-regional shipping service price in US dollars

$\varepsilon_{r,s}$: exchange rates to convert the *r*-th region's currency into the *s*-th region's currency

Exogenous variables and parameters

S_r^f: current account deficits in US dollars

$FF_{h,j,r}$: factor endowment initially employed in the *j*-th sector

$TFP_{j,r}$: productivity; $TFP_{wheat,r} \sim N(1, \sigma_r^2) \text{or} N(1,0)$

σ_r: standard deviation of productivity in wheat sector

$Z_{j,r}^0$: initial amount of gross output

τ_r^d: direct tax rates

$\tau_{i,r}^z$: production tax rates

$\tau_{i,s,r}^m$: import tariff rates on inbound shipping from the *s*-th region

$\tau_{i,r,s}^e$: export tax rates on outbound shipping to the *s*-th region

$\tau_{i,r,s}^{s}$: inter-regional shipping service requirement per unit transportation of the *i*-th good from the *r*-th region to the *s*-th region

$\tau_{h,j,r}^{f}$: factor input tax rates

- Household

(Utility function: $UU_r = XFD_r^{\alpha_r^{XFD}} \prod_{nfd} X_{nfd,r}^{p\ \ \alpha_{nfd,r}} \quad \forall\ r$). (S1)

Demand functions for consumption

$$X_{nfd,r}^{p} = \frac{\alpha_{nfd,r}}{p_{nfd,r}^{q}} \left(\sum_{h,j} p_{h,j,r}^{f} F_{h,j,r} - T_r^d - S_r^p \right) \quad \forall\ nfd,\ r. \tag{S2}$$

$$XFD_r = \frac{\alpha_r^{XFD}}{p_r^{XFD}} \left(\sum_{h,j} p_{h,j,r}^{f} F_{h,j,r} - T_r^d - S_r^p \right) \quad \forall\ r. \tag{S3}$$

Food composite aggregation function

$$XFD_r = \Theta_r \left(\sum_{fd} \Delta_{fd,r} X_{fd,r}^{p\ \Psi} \right)^{1/\Psi} \quad \forall\ r, \tag{S4}$$

(Note that $\Psi = (\varepsilon^f - 1)/\varepsilon^f$).

$$X_{fd,r}^{p} = \left(\frac{\Theta_r{}^{\Psi} \Delta_{fd,r} p_r^{XFD}}{p_{fd,r}^{q}} \right)^{\frac{1}{1-\Psi}} XFD_r \forall\ fd,\ r. \tag{S5}$$

Savings function

$$S_r^p = s_r^p \sum_{h,j} p_{h,j,r}^{f} F_{h,j,r}\ \forall\ r. \tag{S6}$$

- Value added producing firm

Factor demand function

$$F_{h,j,r} = \left(\frac{ {b_{j,r}}^{\eta_j^{va}} \beta_{h,j,r} p_{j,r}^{y} }{ \left(1+\tau_{h,j,r}^{f}\right) \ p_{h,j,r}^{f} } \right)^{\frac{1}{1-\eta_j^{va}}} Y_{j,r} \forall \ h, \ j, \ r, \qquad \text{(S7)}$$

(Note that $\eta_i^{va} = (\varepsilon^{va} - 1)/\varepsilon^{va}$).

Value added production function

$$Y_{j,r} = b_{j,r} \left(\sum_h \beta_{h,j,r} {F_{h,j,r}}^{\eta_j^{va}} \right)^{1/\eta_j^{va}} \ \forall \ j, \ r. \qquad \text{(S8)}$$

- Gross output producing firm

(Production function:

$$Z_{j,r} = TFP_{j,r} \, min \left(\left\{ \frac{X_{i,j,r}}{ax_{i,j,r}} \right\}_i , \frac{Y_{j,r}}{ay_{j,r}} \right) \ \forall \ j, \ r). \qquad \text{(S9)}$$

Demand function for intermediates

$$X_{i,j,r} = \frac{ax_{i,j,r} Z_{j,r}}{TFP_{j,r}} \ \forall \ i, \ j, \ r. \qquad \text{(S10)}$$

Demand function for value added

$$Y_{j,r} = \frac{ay_{j,r} Z_{j,r}}{TFP_{j,r}} \qquad \forall \ j, \ r. \qquad \text{(S11)}$$

Unit price function

$$p_{j,r}^{z} = \frac{1}{TFP_{j,r}} \left(\sum_i ax_{i,j,r} p_{i,r}^{q} + ay_{j,r} p_{j,r}^{y} \right) \qquad \forall \ j, \ r. \qquad \text{(S12)}$$

- Government

Demand function for government consumption

$$X_{i,r}^{g} = \frac{\iota_{i,r}}{p_{i,r}^{q}}\left(T_r^d + \sum_{h,j} T_{h,j,r}^{f} + \sum_j T_{j,r}^{z} + \sum_{j,s} T_{j,s,r}^{m} + \sum_{j,s} T_{j,r,s}^{e} - S_r^g\right) \quad \forall\ i,\ r. \tag{S13}$$

Direct tax revenue

$$T_r^d = \tau_r^d \sum_{h,j} p_{h,j,r}^{f} F_{h,j,r} \qquad \forall\ r. \tag{S14}$$

Production tax revenue

$$T_{j,r}^{z} = \tau_{j,r}^{z} p_{j,r}^{z} Z_{j,r} \qquad \forall\ j,\ r. \tag{S15}$$

Import tariff revenue

$$T_{j,s,r}^{m} = \tau_{j,s,r}^{m}\left[\left(1+\tau_{j,s,r}^{e}\right)\varepsilon_{s,r} p_{j,s,r}^{t} + \tau_{j,s,r}^{s}\varepsilon_{USA,r} p^{s}\right]\ \ T_{j,s,r} \qquad \forall\ j,s,r. \tag{S16}$$

Export tax revenue

$$T_{j,r,s}^{e} = \tau_{j,r,s}^{e} p_{j,r,s}^{t} T_{j,r,s} \qquad \forall\ j,r,s. \tag{S17}$$

Factor input tax revenue

$$T_{h,j,r}^{f} = \tau_{h,j,r}^{f} p_{h,j,r}^{f} F_{h,j,r} \qquad \forall\ h,j,r. \tag{S18}$$

Government savings function

$$S_r^g = s_r^g\left(T_r^d + \sum_{h,j} T_{h,j,r}^{f} + \sum_j T_{j,r}^{z} + \sum_{j,s} T_{j,s,r}^{m} + \sum_{j,s} T_{j,r,s}^{e}\right) \qquad \forall\ r. \tag{S19}$$

- Investment

Demand function for commodities for investment uses

$$X_{i,r}^{v} = \frac{\lambda_{i,r}}{p_{i,r}^{q}}\left(S_r^p + S_r^g + \varepsilon_{USA,r} S_r^f\right) \qquad \forall\ i,\ r. \tag{S20}$$

- Armington composite good producing firm

Composite good production function

$$Q_{i,r} = \gamma_{i,r}\left(\delta^m_{i,r} {M_{i,r}}^{\eta_i} + \delta^d_{i,r} {D_{i,r}}^{\eta_i}\right)^{1/\eta_i} \qquad \forall\ i,\ r, \qquad \text{(S21)}$$

(Note that $\eta_i = (\varepsilon - 1)/\varepsilon$).

Composite import demand function

$$M_{i,r} = \left(\frac{{\gamma_{i,r}}^{\eta_i} \delta^m_{i,r} p^q_{i,r}}{p^m_{i,r}}\right)^{\frac{1}{1-\eta_i}} Q_{i,r} \qquad \forall\ i,\ r. \qquad \text{(S22)}$$

Domestic good demand function

$$D_{i,r} = \left(\frac{{\gamma_{i,r}}^{\eta_i} \delta^d_{i,r} p^q_{i,r}}{p^d_{i,r}}\right)^{\frac{1}{1-\eta_i}} Q_{i,r} \qquad \forall\ i,\ r. \qquad \text{(S23)}$$

- Import variety aggregation firm

Composite import function

$$M_{i,r} = \omega_{i,r}\left(\sum_s \kappa_{i,s,r} {T_{i,s,r}}^{\varpi_i}\right)^{1/\varpi_i} \qquad \forall\ i,\ r. \qquad \text{(S24)}$$

Import demand function

$$T_{i,s,r} = \left(\frac{{\omega_{i,r}}^{\varpi_i} \kappa_{i,s,r} p^m_{i,r}}{(1+\tau^m_{i,s,r})\left[(1+\tau^e_{i,s,r})\varepsilon_{s,r} p^t_{i,s,r} + \tau^s_{i,s,r}\varepsilon_{USA,r} p^s\right]}\right)^{\frac{1}{1-\varpi_i}} M_{i,r} \qquad \forall\ i, s, r. \qquad \text{(S25)}$$

- Gross output transforming firm

CET transformation function

$$Z_{i,r} = \theta_{i,r}\left(\xi^e_{i,r} {E_{i,r}}^{\varphi_i} + \xi^d_{i,r} {D_{i,r}}^{\varphi_i}\right)^{1/\varphi_i} \qquad \forall\ i, r. \qquad \text{(S26)}$$

(Note that $\varphi_i = (\varepsilon_i + 1)/\varepsilon_i$).

Composite export supply function

$$E_{i,r} = \left(\frac{{\theta_{i,r}}^{\varphi_i} \xi^e_{i,r} (1+\tau^z_{i,r}) p^z_{i,r}}{p^e_{i,r}}\right)^{\frac{1}{1-\varphi_i}} Z_{i,r} \qquad \forall\ i, r. \qquad \text{(S27)}$$

Domestic good supply function

$$D_{i,r} = \left(\frac{\theta_{i,r}{}^{\varphi_i}\xi_{i,r}^{d}(1+\tau_{i,r}^{z})p_{i,r}^{z}}{p_{i,r}^{d}}\right)^{\frac{1}{1-\varphi_i}} Z_{i,r} \qquad \forall\ i, r. \tag{S28}$$

- Export variety producing firm

Composite export transformation function

$$E_{i,r} = \varsigma_{i,r}\left(\sum_s \rho_{i,r,s} T_{i,r,s}{}^{\phi_i}\right)^{1/\phi_i} \qquad \forall\ i, r. \tag{S29}$$

Export supply function

$$T_{i,r,s} = \left(\frac{\varsigma_{i,r}{}^{\phi_i}\rho_{i,r,s}p_{i,r}^{e}}{p_{i,r,s}^{t}}\right)^{\frac{1}{1-\phi_i}} E_{i,r} \qquad \forall\ i, r, s. \tag{S30}$$

Balance of payments

$$\sum_{i,s}(1+\tau_{i,r,s}^{e})\,\varepsilon_{r,USA}p_{i,r,s}^{t}T_{i,r,s} + S_r^f + \varepsilon_{r,USA}(1+\tau_{TRS,r}^{z})p_{TRS,r}^{z}TT_r$$
$$= \sum_{i,s}\left[\tau_{i,s,r}^{s}p^{s}\,\varepsilon_{USA,USA} + (1+\tau_{i,s,r}^{e})p_{i,s,r}^{t}\varepsilon_{s,USA}\right]T_{i,s,r} \qquad \forall\ r. \tag{S31}$$

- Inter-regional shipping sector

Inter-regional shipping service production function

$$Q^s = c\prod_r TT_r{}^{\chi_r} \tag{S32}$$

Input demand function for international shipping service provided by the r-th country

$$TT_r = \frac{\chi_r}{(1+\tau_{TRS,r}^{z})\ \ \varepsilon_{r,USA}p_{TRS,r}^{z}}p^sQ^s \qquad \forall\ r. \tag{S33}$$

- Market-clearing conditions

Commodity market

$$Q_{i,r} = X_{i,r}^{p} + X_{i,r}^{g} + X_{i,r}^{v} + \sum_j X_{i,j,r} \qquad \forall\ i,r. \tag{S34}$$

- Capital markets

$$FF_{CAP,j,r} = F_{CAP,j,r} \qquad \forall\ j,r. \tag{S35}$$

Labor market

$$\sum_j FF_{LAB,j,r} = \sum_j F_{LAB,j,r} \qquad \forall\ r. \tag{S36}$$

$$p_{LAB,j,r}^{f} = p_{LAB,i,r}^{f} \qquad \forall\ i,j,r. \tag{S37}$$

Foreign exchange rate arbitrage condition

$$\varepsilon_{r,r'} \cdot \varepsilon_{r',s} = \varepsilon_{r,s} \qquad \forall\ r,r',s. \tag{S38}$$

Inter-regional shipping service market

$$Q^{s} = \sum_{i,r,s} \tau_{i,r,s}^{s} T_{i,r,s}. \tag{S39}$$

- Land allocation

CET transformation function

$$LAND_{r\_sh} = \theta^{ld}{}_{r\_sh}\left(\xi_{r\_sh}^{lsk} LSK_{r\_sh}{}^{\varphi_{ld}} + \xi_{r\_sh}^{crp} CRP_{r\_sh}{}^{\varphi_{ld}}\right)^{1/\varphi_{ld}} \qquad \forall\ i,r. \tag{S40}$$

(Note that $\varphi_{ld} = (\varepsilon^{ld} + 1)/\ \varepsilon^{ld}$).

Livestock supply function

$$LSK_{r\_sh} = \left(\frac{\theta_{r\_sh}^{ld}{}^{\varphi^{ld}} \quad \xi_{r\_sh}^{lsk} p_{r\_sh}^{ld}}{p_{r\_sh}^{lsk}}\right)^{\frac{1}{1-\varphi^{ld}}} LAND_{r\_sh} \qquad \forall\ r\_sh. \tag{S41}$$

Crop supply function

$$CRP_{r\_sh} = \left(\frac{\theta_{r\_sh}^{ld}{}^{\varphi^{ld}} \xi_{r\_sh}^{d} p_{r\_sh}^{ld}}{p_{r\_sh}^{crp}} \right)^{\frac{1}{1-\varphi^{ld}}} LAND_{r\_sh} \qquad \forall\ r\_sh. \tag{S42}$$

CET transformation function for crops

$$CRP_{r\_sh} = \theta^{crp}{}_{r\_sh} \left(\xi_{r\_sh}^{ogc} OGC_{r\_sh}{}^{\varphi_{crp}} + \sum_j \xi_{j,r\_sh}^{ocrp} OCRP_{j,r\_sh}{}^{\varphi_{crp}} \right)^{1/\varphi_{crp}} \qquad \forall\ r\_sh. \tag{S43}$$

(Note that $\varphi_{ld} = (\varepsilon^{crp} + 1)/\ \varepsilon^{crp}$).

Aggregated oil-crops and grain supply function

$$OGC_{r\_sh} = \left(\frac{\theta_{r\_sh}^{crp}{}^{\varphi^{crp}} \xi_{r\_sh}^{ogc} p_{r\_sh}^{crp}}{p_{r\_sh}^{ogc}} \right)^{\frac{1}{1-\varphi^{crp}}} CRP_{r\_sh} \qquad \forall\ r\_sh. \tag{S44}$$

Other crops supply function

$$OCRP_{j,r\_sh} = \left(\frac{\theta_{r\_sh}^{crp}{}^{\varphi^{crp}} \xi_{j,r\_sh}^{ocrp} p_{r\_sh}^{crp}}{p_{r\_sh}^{ocrp}} \right)^{\frac{1}{1-\varphi^{crp}}} CRP_{r\_sh} \qquad \forall\ r\_sh. \tag{S45}$$

CET transformation function for oil crop-grain composite

$$OGC_{r\_sh} = \theta^{ogc}{}_{r\_sh} \sum_j \left(\xi_{j\_og,r\_sh}^{ogc} F_{j\_og,r\_sh}{}^{\varphi_{ogc}} \right)^{\frac{1}{1-\varphi^{ogc}}} \qquad \forall\ r\_sh. \tag{S46}$$

(Note that $\varphi_{ogc} = (\varepsilon^{ogc} + 1)/\ \varepsilon^{ogc}$).

Oil crops and grain supply function

$$F_{j\_og,r\_sh} = \left(\frac{\theta_{r\_sh}^{ogc}{}^{\varphi^{ogc}} \xi_{jog,r\_sh}^{ogc} p_{r\_sh}^{crp}}{p_{jog,r\_sh}^{ogc}} \right)^{\frac{1}{1-\varphi^{ogc}}} CRP_{r\_sh} \qquad \forall\ j\_og, r\_sh. \tag{S47}$$

Supplementary References

Armington, P. S. A theory of demand for products distinguished by place of production. *International Monetary Fund Staff Pap*. 16, 159-178 (1969).

Bajzik, J., Havranek, T., Irsova, Z., & Schwarz, J., Estimating the Armington elasticity: The importance of study design and publication bias, J Int Econ 127 (2020).

FAO, FAOSTAT database (FAO, Rome, Italy, 2021), available at: http://www.fao.org/faostat/en/#data/QC.

Haile, M.G., Brockhaus, J., Kalkuhl, M., Short-term acreage forecasting and supply elasticities for staple food commodities in major producer countries. Agric. Food Econ 4:17 (2016)

Seale, J., Regmi, A., & Bernstein, J., International evidence on food consumption patterns. United States Department of Agriculture Technical Bulletin Number 1904 (2003).

Timilsina, G.R., Beghin, J.C., Mensbbrugghe, D., Mevel, S. The impacts of biofuels targets on land-use change and food supply: A global CGE assessment. Agr Econ 43, 315—332 (2012)

USDA (2023), The World Agricultural Supply and Demand Estimates (WASDE). Source URL: https://www.usda.gov/oce/commodity/wasde.
